# Genome-Wide Identification and Characterization of SNPs and InDels of *Capsicum annuum* var. *glabriusculum* from Mexico Based on Whole Genome Sequencing

**DOI:** 10.3390/plants13223248

**Published:** 2024-11-20

**Authors:** Valeria Itzel Moreno-Contreras, Ma. Carmen E. Delgado-Gardea, Jesús A. Ramos-Hernández, Alfonso Mendez-Tenorio, Hugo Varela-Rodríguez, Blanca Sánchez-Ramírez, Zilia Y. Muñoz-Ramírez, Rocío Infante-Ramírez

**Affiliations:** 1 Facultad de Ciencias Químicas, Universidad Autónoma de Chihuahua, Campus II Circuito Universitario s/n, Chihuahua 31125, Mexico; 2 Laboratorio de Biotecnología y Bioinformática Genómica, Departamento de Bioquímica, Escuela Nacional de Ciencias Biológicas, Instituto Politécnico Nacional, Campus Lázaro Cárdenas, Mexico City 11340, Mexico; 3 Facultad de Medicina y Ciencias Biomédicas, Universidad Autónoma de Chihuahua, Campus II Circuito Universitario s/n, Chihuahua 31125, Mexico

**Keywords:** bioinformatics, chili pepper, variant calling, whole genome sequencing, molecular markers

## Abstract

*Capsicum annuum* var. *glabriusculum* is an economically important horticultural crop and is considered the wild genetic ancestor of chili peppers. The distribution range extends from southern North America, through Central America, to South America. Approximately 226 million 150 paired-end reads were generated from CHMX_Ch1 (a *C. annuum* from Chihuahua, Mexico). To compare with the CHMX_Ch1 genome, high-quality reads from QO (a *C. annuum* from Querétaro, Mexico) were downloaded from the NCBI database. A total of 210,324 variants were detected in CHMX_Ch1, whereas 169,718 variants were identified in QO, all compared to the domesticated *C. annuum* reference genome, UCD10Xv1.1. This comprised 203,990 SNPs and 6334 InDels in CHMX_Ch1 and 164,955 SNPs and 4763 InDels in QO. The variants with high and moderate impact were identified as missense, splice acceptor, splice donor, start lost, stop gain, stop lost, frameshift, insertion, and deletion effects. The candidate genes with the highest fold enrichment values among the SNPs were predominantly involved in gene regulation and metabolic processes. InDels were associated with nuclear and transcriptional regulator activity in both genomes. Overall, a greater number of variants were found in CHMX_Ch1 compared to QO. This study provides knowledge of the principal functions associated with high- and moderate-impact variants and supplies a resource for further investigations of the genetic characteristics of these chiltepin peppers.

## 1. Introduction

The fruit of *Capsicum*, commonly known as chili pepper, has significant economic importance as a versatile horticultural crop, with applications in nutrition, pharmaceuticals, recreation, and ornamental purposes [1,2,3,4,5,6,7,8,9,10,11,12,13,14,15]. Within the *Capsicum* genus, which comprises over 30 species, *C. annuum* stands out as the most globally cultivated variant, exhibiting a diverse array of fruit colors, sizes, and shapes [2,9,16,17,18,19,20]. To harness the full potential of the *Capsicum* species, which have demonstrated adaptability to varying climatic conditions, diseases, and soil characteristics, it becomes imperative to deepen our understanding of the genetic diversity within *Capsicum* populations [9,15,17,21,22,23,24].

The chiltepin pepper (*Capsicum annuum* var. *glabriusculum*), identified as the wild genetic ancestor of domesticated chili peppers (*C. annuum*), presents a unique subset within the *Capsicum annuum* species [25,26,27,28,29,30]. Notably, certain populations exhibit a wild, semiwild, or semidomesticated status and thrive in tropical and semiarid environments, spanning from the southern United States of America to South America. Particularly well suited for growth in hot and dry climates, the chiltepin pepper presents remarkable phenotypic diversity, which is evident in significant variations in leaf and fruit morphology among different varieties and cultivars [25,26,31,32]. Furthermore, disparities in seed germination and susceptibility to diseases are notable across various populations, with these effects potentially exacerbated by environmental conditions, posing challenges to the development of effective agricultural strategies. Despite these obstacles, the high genetic diversity inherent in the chiltepin pepper makes it a valuable genetic resource for advancing the cultivation of pepper crops [30,33,34,35,36].

The rapid advancement of next-generation sequencing (NGS) technologies, particularly in the realm of genomics, has provided researchers with unprecedented opportunities to delve into the entire genome of chili peppers. This approach has enabled meticulous decoding of genetic functions and unraveling of the molecular underpinnings across diverse crop members, shedding light on the causal factors behind phenotypic changes [2,37,38]. In recent years, the genomes of domesticated and wild *Capsicum* species have been sequenced and released. The first whole genome sequencing and assembly were conducted on the Mexican genomes of *C. annuum* cv. Criollo de Morelos 334 (known as CM334) and *C. chinense* PI159236. Both genomes have been widely used as founders of mapping populations because of their resistance to various diseases and pests [6,24,39]. The genomes of *C. annuum* Zunla-1 and *C. annuum* var. *glabriusculum* were subsequently sequenced, providing valuable insights into the evolution of this plant and the Solanaceae family [9,39,40]. This study revealed that the expansion of the size of the hot pepper genome is due to transposable elements. Additionally, a list of candidate genes involved in domestication syndrome was provided [9].

In this study, we aimed to identify variations (SNPs and InDels) between the genomes of two types of peppers: *C. annuum* var. *glabriusculum* (CHMX_Ch1), from Chihuahua, Mexico, and *C. annuum* var. *glabriusculum* (QO), from Queretaro, Mexico. These specific varieties were chosen not only due to their distinct geographical origins but also because they may exhibit unique genetic adaptations to different environmental conditions. Understanding the genetic differences between CHMX_Ch1 and QO can provide insights into traits related to stress tolerance, disease resistance, and other factors crucial for pepper adaptability and cultivation in varying climates. The SNPs and InDels were categorized into different metabolic pathways, and genes associated with pathways that could influence plant adaptability were described. This research represents the first comparative genome analysis of Mexican chili genomes, offering a comprehensive report on the identification and association of genome-wide SNPs and InDels with plant adaptability. The findings provide valuable knowledge and resources for further investigations of the genetic characteristics of these chiltepin peppers to increase the quality of this plant.

## 2. Results

### 2.1. Preprocessing Reads and Mapping of CHMX_Ch1 and QO to the Reference Genome

In total, 262,995,253 paired-end raw reads were obtained for the CHMX_Ch1 genome, with a mean length of 150 bp. After the removal of adaptor sequences and ambiguous and low-quality reads, a total of 262,921,020 reads were retained.

To compare the CHMX_Ch1 genome, high-quality reads from the other *C. annuum* var. *glabriusculum* genome [9], sequenced from Queretaro, Mexico (referred to as QO in this analysis), were downloaded from the NCBI database (SRP018258). A total of 513,752,953 (96.98%) and 443,277,983 (98.37%) high-quality reads of CHMX_Ch1 and QO, respectively, were successfully mapped to the reference genome. The reference genome used in this study was *C. annuum* UCD10Xv1.1 (GCF_002878395.1), which is well-characterized and commonly used for comparative studies due to its high-quality assembly [41]. This genome serves as a benchmark to identify genetic variations in CHMX_Ch1 and QO.

### 2.2. Genome-Wide Identification and Characterization of SNPs and InDels

**Figure 1:**
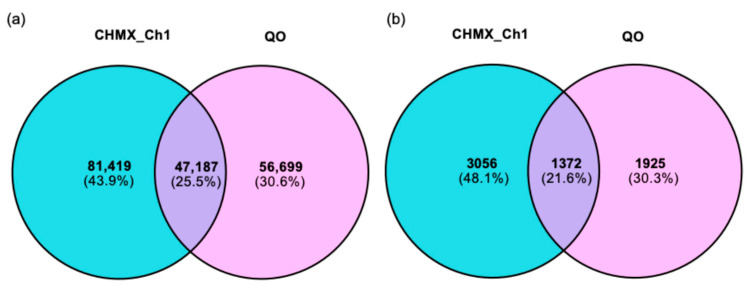
Venn diagrams displaying genomic variations in the CHMX_Ch1 and QO genomes compared to reference genome. (**a**) SNP distribution comparison; (**b**) InDel distribution comparison.

The distribution of SNPs/InDels detected between CHMX_Ch1 and QO was analyzed with the reference genome. A total of 203,990 and 164,955 SNPs, as well as 6364 and 4763 InDels, were identified in CHMX_Ch1 and QO, respectively, when compared to the reference genome. The average density of SNPs and InDels in CHMX_Ch1 was approximately 196.93 SNPs/Mb and 178.07 InDels/Mb, whereas in QO, it was 197.26 SNPs/Mb and 184.16 InDels/Mb. SNPs’ and InDels’ densities for each chromosome are presented in Table S1. The number of unique SNPs detected in CHMX_Ch1 and QO was 81,419 (43.9%) and 56,699 (30.6%), respectively. In total, 47,187 (25.5%) SNPs were found to be common in both genomes. On the other hand, a total of 3056 (48.1%) InDels were detected in CHMX_Ch1, whereas 1925 (30.3%) were detected in QO. Only 1372 (21.6%) InDels were identified in common between the two genomes (Figure 1).

**Figure 2:**
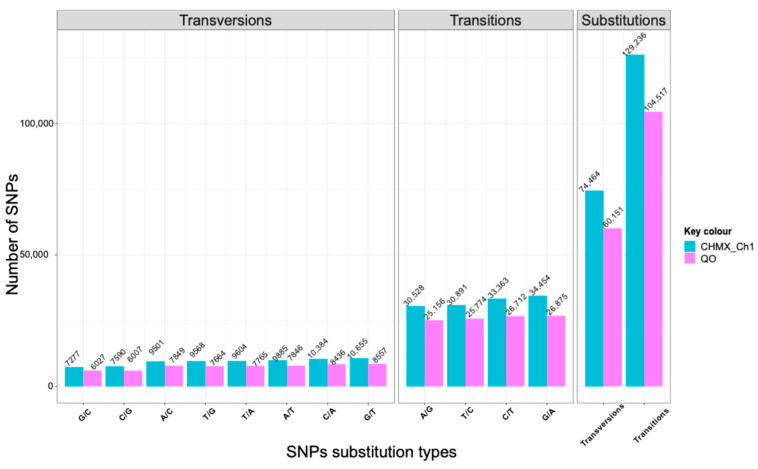
Transition (Ts) and transversion (Tv) frequencies of the SNPs in CHMX_Ch1 and QO.

CHMX_Ch1 and QO had Ts/Tv ratios of 1.73 and 1.73, respectively, for transition and transversion SNPs. The most frequent types of SNP transitions were G/A and C/T for both genomes. The frequencies of transversions were less frequent and similar in both genomes (Figure 2).

**Figure 3:**
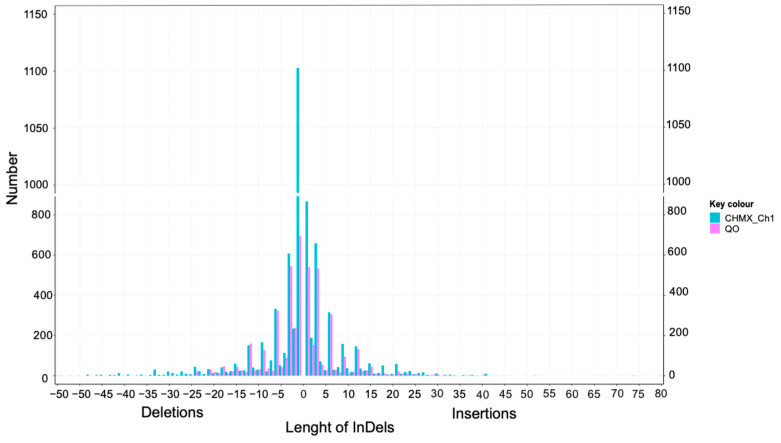
Frequency of the length distribution of InDels in the CHMX_Ch1 and QO pepper genomes.

Insertions and deletions of length observed in CHMX_Ch1 ranged from 1 to 80 bp and 1 to 55 bp, respectively, whereas ranges of 1–66 bp and 1–34 bp, respectively, were observed for QO. The frequency of InDels decreased with increasing length. Nevertheless, for InDels of the same length, CHMX_Ch1 exhibited a greater abundance than the QO genome (Figure 3).

### 2.3. Distribution of SNPs and InDels

**Figure 4:**
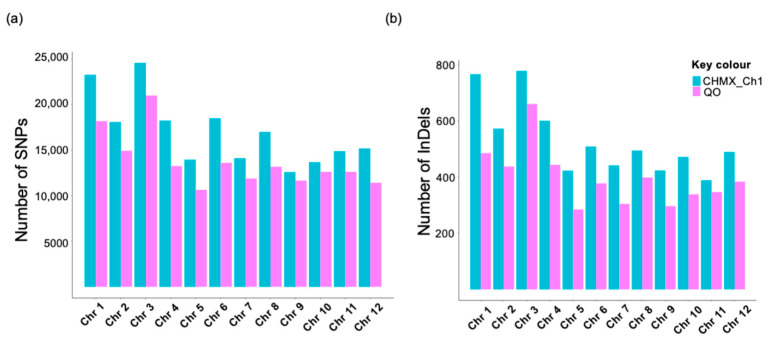
Chromosomal distribution of SNP and InDel numbers in CHMX_Ch1 and QO genomes. (**a**) Number of SNPs across chromosomes. (**b**) Number of InDels across chromosomes.

**Figure 5:**
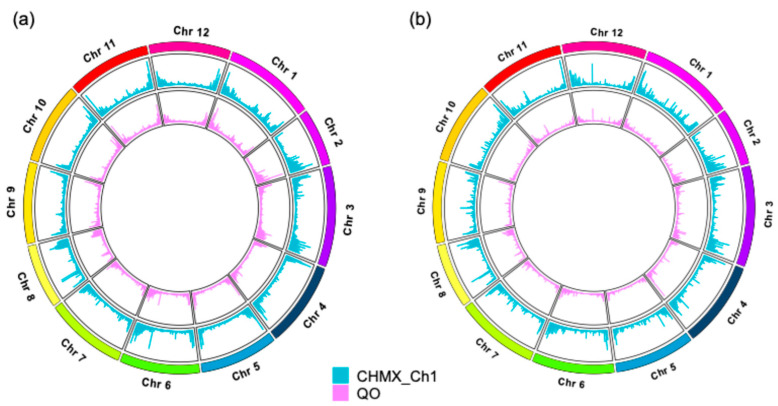
Distribution of genomic variation density across chromosomes in the CHMX_Ch1 and QO genomes. (**a**) SNP density distribution. (**b**) InDel density distribution.

The nonuniform distributions of SNPs and InDels were analyzed across 12 chromosomes in both genomes (Figure 4). Chromosome 3 was the largest and presented the highest frequency of SNPs in both genomes, with 24,567 SNPs in CHMX_Ch1 and 20,980 SNPs in QO. Chromosome 9 had the lowest number of SNPs in CHMX_Ch1, with 12,583 SNPs, while QO showed the lowest number in Chromosome 5, with 10,644 SNPs (Figure 4a). On the other hand, CHMX_Ch1 and QO exhibited the highest number of InDels in Chromosome 3, with 779 and 661 InDels, respectively. The lowest number of InDels was detected in Chromosome 11 for CHMX_Ch1, with 389 InDels, whereas Chromosome 5 displayed the lowest count in QO, with 285 InDels observed (Figure 4b). Furthermore, in both organisms, the most densely distributed variation was observed toward the ends of the chromosomes, as shown in Figure 5.

### 2.4. Effects of Mutations

**Figure 6:**
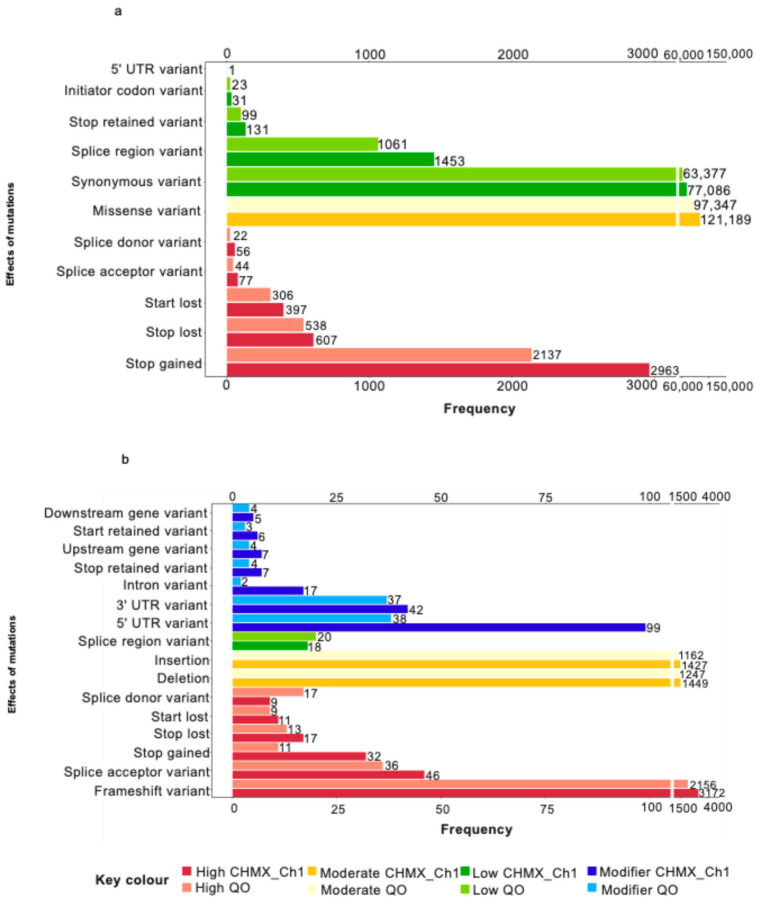
Predicted variant impact analysis in the CDS Regions of CHMX_Ch1 and QO genomes. (**a**) Distribution and predicted impact of SNPs. (**b**) Distribution and predicted impact of InDels.

Predicted amino acid substitutions were analyzed to estimate the potential impact and effect of SNPs and InDels in coding sequences. The number of SNPs and InDels was classified into high, moderate, low, and modifier impacts. In the CHMX_Ch1 and QO genomes, 4100 SNPs (2%) and 3047 SNPs (1.84%) were classified as high impact, including stop gained, stop lost, start lost, splice acceptor, and splice donor variants. A total of 121,189 SNPs (59.40%) and 97,347 SNPs (59.01%) were missense variants grouped into the moderate impact category. The low impact class consisted of 78,701 SNPs (38.58%) and 64,560 SNPs (39.13%), which included synonymous, splice region, stop retained, and initiator codon variants. Additionally, QO exhibited 1 SNP (6 × 10^−4^%) in the 5′ UTR region, classified under the modifier impact category (Figure 6a). In both genomes, the moderate impact category was predominantly associated with nonsynonymous coding variants, specifically missense variants.

In addition, a total of 3287 InDels (51.64%) in CHMX_Ch1 and 2242 InDels (47.07%) in QO were detected in high impact categories, including frameshift, splice acceptor, splice donor, stop lost, stop gained, and start lost variants. Meanwhile, 2876 InDels (45.19%) in CHMX_Ch1 and 2409 InDels (50.57%) in QO were identified as deletions and insertions with moderate impact. Furthermore, the low impact class was represented by splice region variants, with 18 InDels (0.28%) in CHMX_Ch1 and 20 InDels (0.41%) in QO. Additionally, 183 InDels (2.87%) in CHMX_Ch1 and 92 InDels (1.93%) in QO, including 3′ UTR, 5′ UTR, downstream gene, intron, start retained, stop retained, and upstream gene variants, were classified under the modifier impact class (Figure 6b). In both genomes, moderate-impact SNPs were more common than low- and high-impact SNPs were, but the reverse trend was observed for low- and high-impact InDels.

### 2.5. Gene Ontology Analysis and Annotation of High- and Moderate-Impact Genetic Variants

**Figure 7:**
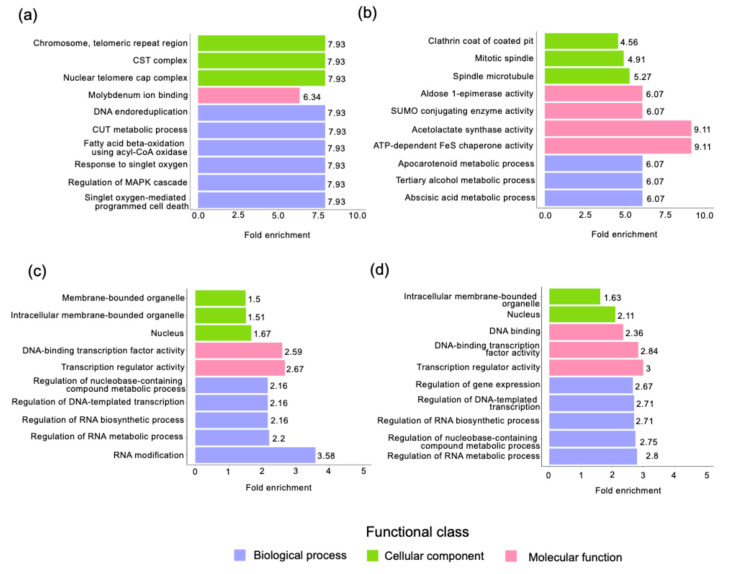
Gene Ontology analysis of the principal genes associated with high and moderate impact detected within the CDS regions of the SNPs in the (**a**) CHMX_Ch1, (**b**) QO, and InDel (**c**) CHMX_Ch1 and (**d**) QO genomes.

The SNPs and InDels with moderate and high impacts were analyzed via PANTHER v18.0 to identify the genes and functions involved. The results of the candidate gene association analyses for the SNPs and InDels were obtained according to biological process, molecular function, and cellular component as functional classes from Gene Ontology, with the highest fold enrichment (FE) values shown in Figure 7. The SNPs with the highest FE in CHMX_Ch1 were involved in DNA endoreduplication, the cryptic unstable transcripts (CUTs) metabolic process, fatty acid beta-oxidation, the response to singlet oxygen, and the regulation of the MAPK cascade (Figure 7a), whereas the SNPs found in the QO genome were involved in different metabolic processes (Figure 7b). The class of cellular component was described by genes in the telomeric repeat region of chromosomes, in the CST complex and nuclear telomere cap complex, which were grouped into the cellular component of GO for the CHMX_Ch1 genome (Figure 7a); moreover, the QO genes presented different characteristics, such as spindle microtubules, mitotic spindles, and clathrin coat pits (Figure 7b). In contrast, the molecular function class CHMX_Ch1 was related to genes involved in molybdenum ion binding (Figure 7a), although the QO genome reflected a close relationship between acetolactate synthase and ATP-dependent FeS chaperone activities (Figure 7b).

On the other hand, the remaining InDels were associated with a greater FE in genes associated with biological processes related to RNA modification in CHMX_Ch1 (Figure 7c) and the regulation of RNA metabolic processes in QO (Figure 7c). In the cellular component category, the InDels included genes associated with binding to the nucleus and intracellular membrane-bound organelles in both CHMX_Ch1 and QO (Figure 7c,d). Notably, both genomes showed molecular functions related to transcription regulator activity and DNA-binding transcription factor activity (Figure 7c,d). All GO enrichment results are available in Table S2.

## 3. Discussion

Previous studies have performed whole genome sequencing of various *Capsicum annuum* varieties to identify associations between SNPs/InDels and diverse traits, including biotic stress, domestication, seed composition, seed size, the seed coat, and flowering [42]. This study extends these analyses by focusing on whole genome sequencing of *Capsicum annuum* var. *glabriusculum* from Chihuahua, Mexico, providing new insights into the genetic basis of adaptations to distinct climatic conditions compared with those found in Querétaro, Mexico. The southwestern region of Chihuahua, Mexico, is characterized by warm subhumid climatic conditions, with an average annual precipitation of 781.7 mm, with forests [43,44]. Moreover, the QO pepper population was obtained near the municipality of El Patol, Queretaro, Mexico [9], which has a dry and semidry climate with an average annual rainfall of 593.9 mm and a mean annual temperature of 20.36 °C [45]. CHMX_Ch1 was selected because of its low germination rate when it was subjected to citric acid (0.45%) for 48 h, in contrast with other pepper varieties, which use scarification and gibberellic acid for 24 h as a pregermination treatment to increase germination rates [46]. The contrasting germination treatments between CHMX_Ch1, which uses citric acid, and other pepper varieties, which use scarification and gibberellic acid, highlight potential genetic adaptations to environmental stressors that warrant further investigation. According to the map alignment, the percentage of high-quality reads mapped to the UCD10X genome was 98.37%, which was greater than that of the CHMX_Ch1 genome (96.98%). Nonetheless, these results are similar to those of other studies, which used CM334, a *Capsicum annuum* cultivar known for its disease resistance traits, as a reference genome for four Italian sweet pepper landraces, with mapping rates ranging from 98.11% to 99.10% [2]. This may be due to UCD10x having the highest-quality pepper genome sequence available [41]. Furthermore, the analysis of SNPs and InDels within our targeted CDS regions revealed significant genomic diversity, with a lower count of variants compared with studies focusing on intergenic regions [47,48,49]. This suggests a concentration of functionally relevant mutations within gene-coding areas that are pertinent to adaptations and phenotypic traits. The number of SNPs and InDels detected in the CHMX_Ch1 and QO genomes was less than that reported in other studies, possibly because of a target for CDS analysis. In other studies, a range from 16,033,452 to 17,389,251 SNPs was found in four Italian *C. annuum* cultivars [2]. On the other hand, whole genome resequencing data from the *C. annuum* cultivars Saengryeg 211 and 82PR66 revealed that 6,695,385 and 4,212,078 SNPs, respectively, were located in intergenic regions, whereas 39,955 and 30,022 SNPs, respectively, were detected in CDS regions [47]. Moreover, the *C. baccatum* PRH1 (a powdery mildew line) and *C. annuum* Saengryeg (a PM-susceptible line) genomes were re-sequenced via SNP calling, and 6,213,009 SNPs (150,932 SNPs in the CDS) and 6,840,889 (39,955 in the CDS) SNPs were identified [4]. In comparison, the study of InDel markers for *C. annuum* cv. G29 and *C. frutescens* cv. PBC688 by whole genome sequencing using *C. annuum* cv. CM334 as the reference genome revealed that the distribution of InDels was commonly located within intergenic regions, identifying a total of 1,664,770 and 533,523 InDels, respectively, while 2519 and 1019 InDels were found in the CDS, respectively [48]. Despite these variants being more prevalent in intergenic regions in plants [49], this could be due to transposable elements, which are mostly associated with the expansion of plant genomes [50]. However, CDSs were maintained for further studies to determine their effects on gene expression levels under different climatic conditions [51].

Venn diagrams were used to assess the similarities and unique variants detected in the CHMX_Ch1 and QO genomes. CHMX_Ch1 accounted for 25.5% of the unique SNP markers and 21.6% of the unique InDel markers, respectively. Overall, the CHMX_Ch1 genome presented the greatest number of unique variants, highlighting the genetic distinctiveness of these populations, which could be leveraged in breeding programs for stress resistance and climatic adaptability. The analysis of similarity and shared variants has been conducted in other studies, such as soybean [52], mango [53], and cattle breeds, to determine the ancestral relationships among different genomes and the selection of variants for additional research [54].

Additionally, the predominant SNP transitions (G/A and C/T) observed in this study align with findings in other plant genomes where transitions are more frequent than transversions [52]. The high frequency of transitions over transversions may be explained by the requirement of changes in the double DNA strand resulting from the conservation of the DNA structure by natural selection [55,56]. InDels have been reported as the second most abundant molecular markers in genomes [57,58]. Through the assessment and identification of InDel markers in *C. annuum* cv. G29 and *C. frutescens* cv. PBC688, the length of InDels ranged from 1 to 49 bp, with most InDels being less than 10 bp in length [48]. Numerous studies mention that the length of InDels is linked to the impact and effects of structural changes in a protein because InDels involving two or more amino acids have a significant effect compared with a single InDel [59]. Although most InDels found in this project were small, other authors have proposed that most of the InDels in protein-coding genes are small, encoding one to five amino acids, and are in the loops of protein structures involved in adaptation. In addition, most small InDels participate in essential proteins [60].

In this study, the number of SNPs and InDels was analyzed across each chromosome. Our results indicate that chromosomes 1 and 3 contain the highest number of variants in both genomes, which is consistent with findings from previous studies. Through whole genome resequencing of four Italian sweet and hot pepper genomes, the greatest number of SNPs was found on chromosome 1 when *C. annuum* cv. CM334 was used as the reference genome [17]. Moreover, in a study of the resequencing of C. frutescens cv. PBC688 and *C. annuum* cv. G29 employing *C. annuum* cv. CM334 as a reference genome, the greatest number of InDels was identified on chromosomes 3 and 11, respectively [48]. This might be because the chromosomes of *C. annuum* var. glabriusculum are diverse in length and heterochromatin content [61]. The average SNP density observed in this study is consistent with previous findings. For instance, the genome assembly of *C. annuum* cv. Takanotsume reported a mean density of 100–200 SNPs per 1 Mb region [62].

In contrast, when comparing the InDels’ density to this work, other authors reported a higher average InDel density in *C. annuum* cv. G29 and *C. frutescens* cv. PBC688, with 604.6 InDels per Mb and 193.8 InDels per Mb, respectively. In CHMX_Ch1 and QO, InDel densities were more moderate, with values ranging from 139.85 to 210.82 InDels/Mb in CHMX_Ch1 and from 139.52 to 256.53 InDels/Mb in QO. For example, the highest InDel density in CHMX_Ch1 was observed on chromosome 3, whereas in QO, it was on chromosome 4. In comparison, *C. frutescens* PBC688 consistently displayed elevated InDel densities across chromosomes, particularly on chromosome 2 with 655.5 InDels/Mb, suggesting regions of increased genetic activity or selection. Meanwhile, *C. annuum* G29 showed its highest InDel density on chromosome 11 (318.8 InDels/Mb), indicating potential loci under selective pressure in domesticated varieties [48].

The variant density in CHMX_Ch1 and QO showed the highest accumulation at the ends of each chromosome, whereas the lowest density was in centromeric regions, as has been reported in sorghum [63]. These results suggest that regions with low-density variants may be highly conserved sequences [64].

The variants were annotated to provide insight into the potential functional effects of the coding proteins. The most common mutations in the SNP markers were predicted missense variants with moderate impact, whereas frameshift variants with high impact and insertions and deletions with moderate impact were predominant in the InDel markers in the CHMX_Ch1 and QO genomes. In other studies, the annotation process revealed that the most prevalent type of effect occurs in intergenic regions, with a modifier impact [65,66]. Nonetheless, several studies revealed that nonsynonymous mutations such as missense variants were found at a greater rate in wild ancestral plants than in cultivars, suggesting their influence on the domestication process [67,68]. This phenomenon could be explained by the removal of deleterious amino acid mutations and the conservation of neutral variants over time [69]. Domestication syndrome has been described as a plant breeding strategy to enhance fruits and seeds, along with a decrease in secondary metabolites [70].

Similar to earlier investigations in cattle breeds [71], the variants defined with high impact and moderate impact were evaluated for their potential affections in coding proteins [72]. Thus, the functional annotation of genes detected in variants was linked to diverse pathways, providing a resource for further analysis. In the present work, the candidate gene list revealed that the SNPs with the highest FE in CHMX_Ch1 were genes related to the signaling of proteins in cellular organelles, metabolic processes, and gene regulation; at the same time, the QO genome revealed that genes were mostly linked to diverse metabolic processes. In previous studies, SNPs were shown to be involved primarily in carbohydrate metabolism, ion binding, transcription regulation, and nucleotide binding [4]. In contrast, the functional annotation of genes via GO revealed that most of the genes were associated with RNA modification in the CHMX_Ch1 and QO genomes, whereas in past reports in *C. annuum* cv. G29 and *C. frutescens* cv. PBC688, the InDel markers associated with GO performance were associated with genes involved in metabolic processes, cellular processes, the response to stimulus, and catalytic activity [48].

This comprehensive genomic analysis not only enhances our understanding of *Capsicum* genetic diversity but also provides a foundation for future studies aimed at correlating specific genetic variations with phenotypic characteristics. By doing so, it is possible to advance our knowledge of plant adaptation mechanisms, contributing to the development of cultivars optimized for specific environmental conditions.

## 4. Materials and Methods

### 4.1. Plant Material and DNA Extraction

The plant material of the chiltepin pepper *Capsicum annuum* var. *glabriusculum* was collected from the municipality of Chinipas, Chihuahua (Location: 27°24′0″ N, 108°32′0″ W; 555 masl). Thus, the seeds were extracted from the fruits, placed in a Petri dish, and incubated for 48 h in 0.45% citric acid to break seed dormancy. Therefore, the sprouts were cultivated for six weeks to obtain plants. The plants exhibited several distinctive traits, including a rough texture on the seed surface, dense pubescence, deltoid-shaped leaves, and an almost round fruit shape. Detailed morphological characteristics of the plants under study are available in Table S3 and Figure S1. Total genomic DNA was extracted from 200 mg of young leaves collected from a single plant following a modified CTAB method [73]. The resulting DNA was subsequently purified via a DNA Clean-up and Concentration Zymo Kit (Zymo Research, Irvine, CA, USA) following the manufacturer’s instructions. The quality and integrity of the DNA were quantified via a NanoDrop 2000c spectrophotometer (Thermo Scientific, Wilmington, DE, USA) on the basis of the A260/280 ratio, and the results were observed via 1.0% agarose gel electrophoresis.

### 4.2. Library Preparation and Whole Genome Sequencing

The preparation and sequencing of DNA were carried out at Novogene (Beijing, China) via an Illumina sequencer (Illumina Inc., San Diego, CA, USA). The DNA was randomly fragmented into approximately 350 bp pieces and used for library construction using the NEBNext^®^ DNA Library Prep Kit (New England Biolabs, Ipswich, MA, USA), in strict accordance with the provided instructions. Following purification and quality assessment, the prepared library was sequenced. Hiq 2000 sequencing was used alongside a paired-end 2 × 150 bp strategy. Reads of low quality, those containing adaptor sequences, and duplicates were filtered out. The remaining high-quality data were then employed for further analysis.

### 4.3. SNP/InDel Calling and Variant Annotation

The genome sequences of *Capsicum annuum* var. *glabriusculum* from Queretaro, Mexico, were downloaded from the NCBI database (SRP018258). In this study, the sequences of this genome were identified as QO. The sequences of the CHMX_Ch1 and QO reads were aligned to the latest version of the UCD10Xv1.1 reference genome (GCF_002878395.1) [41] using BWA v0.7.17 [74] and the ‘mem’ command with the default parameters except for the minimum seed length (k = 32). BAM files were processed and used for SNP and InDel calling using SAMtools v1.7 [75] mpileup and filter programs with default parameters except for minimum mapping quality (Q = 100). SNPs/InDels were counted and analyzed using custom bash scripts. The identified genomic variants were analyzed using the SnpEff v5.1 program [76] to deduce their functional annotations and assess any potential deleterious impacts on protein structure.

According to SnpEff v.5, each SNP/InDel is categorized into one of four classes of impact: (1) high, for variants that cause changes in frameshift changes by modifying splice sites and inducing or removing stop codons; (2) moderate, for variants that alter the amino acid sequence; (3) low, representing synonymous variants in coding regions; and (4) modifier, for variants situated outside genes (non-transcribed regions or introns). Only high- and moderate-impact variants were retained for further analysis. Finally, the positions of the gene variants were compared to the reference genome’s annotation with the BEDtools v2.31.1 [77] intersect command.

### 4.4. Gene Ontology Analysis

The gene IDs from high- and moderate-effect variants were obtained to perform a Gene Ontology (GO) analysis with PANTHER v18.0 [78], which specifies a Fisher’s exact test with a false discovery rate correction (FDR) < 0.05 to identify statistically overrepresented functional classes associated with biological processes, molecular functions, and cellular components, with the *C. annum* gene list used as a reference.

## 5. Conclusions

This study performed whole genome sequencing on the chiltepin pepper CHMX_Ch1, conducting a comparative genomic analysis with the QO variety using SNPs and InDels. A higher frequency of variants, primarily nonsynonymous SNPs and frameshift InDels, was observed in CHMX_Ch1, suggesting distinct environmental adaptations between the variants. These differences offer opportunities for developing targeted breeding strategies to increase traits such as stress resistance and yield.

Preserving wild pepper varieties such as chiltepin is crucial, as they harbor genetic resources that can improve cultivated peppers. Future research should focus on differential gene expression linked to these variants and include other chiltepin populations to further understand their genetic architecture.

The advanced sequencing techniques used in this study represent significant progress in crop genomics, supporting the sustainable use and conservation of *Capsicum* biodiversity. These insights provide a foundation for enhancing chili pepper breeding through biotechnological approaches and addressing global agricultural challenges.

## Data Availability

The raw whole genome sequencing sequences are available at the short-read archive (SRA) of the NCBI under the BioProject accession number PRJNA947116. All other data associated with the current study are available from the corresponding author upon reasonable request.

## Supplementary Materials

The following supporting information can be downloaded from https://www.mdpi.com/article/10.3390/plants13223248/s1, Figure S1: Morphological characteristics of *C. annuum* var. *glabriusculum* from Chinipas, Chihuahua, Table S1: InDels and SNPs densities identified on individual chromosomes, Table S2: Gene Ontology enrichment analysis of high- and moderate-impact SNPs and InDels in the CHMX_Ch1 and QO Genomes, Table S3: Morphological characteristics of *C. annuum* plants (CHMX_Ch1 and QO).
