# Supplementary material for: Genome-Wide Identification and Characterization of SNPs and InDels of Capsicum annuum var. glabriusculum from Mexico Based on Whole Genome Sequencing
Source: Plants (Basel). 2024 Nov 20;13(22):3248. doi: 10.3390/plants13223248 (PMC11597950; doi:10.3390/plants13223248)
Supplement: Supplementary file 1 [file plants-13-03248-s001.zip › FigureS1.pdf]

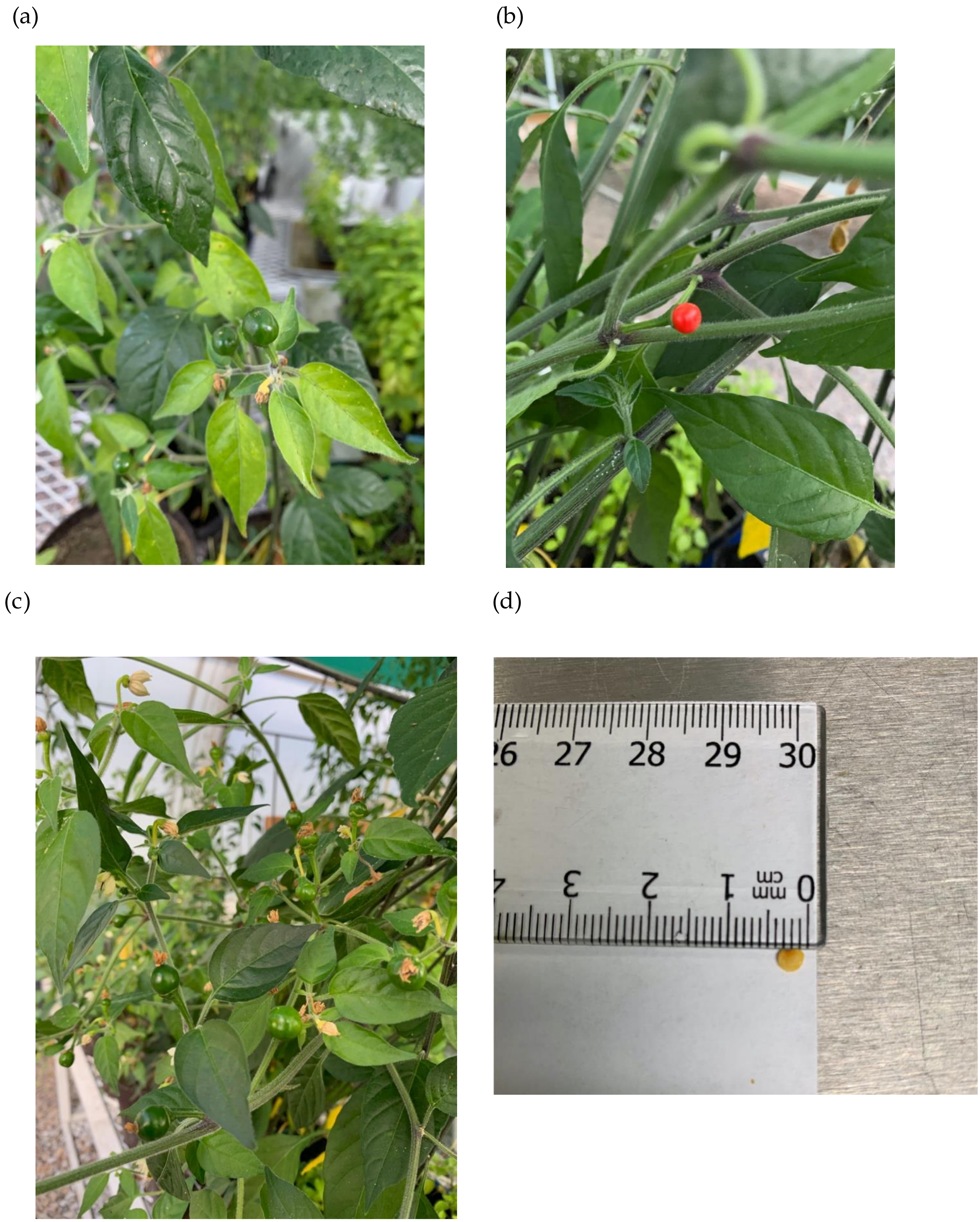

Figure S1. Morphological characteristics of *C. annuum* var. *glabriusculum* from Chinipas, Chihuahua. (a) General view of the plant with deltoid-shaped leaves; (b) Close-up of a mature fruit; (c) Plant showing dense pubescence and clusters of immature fruits; (d) Seed measurement illustrating rough seed texture and size.
