# Supplementary material for: Genome-Wide Identification and Characterization of SNPs and InDels of Capsicum annuum var. glabriusculum from Mexico Based on Whole Genome Sequencing
Source: Plants (Basel). 2024 Nov 20;13(22):3248. doi: 10.3390/plants13223248 (PMC11597950; doi:10.3390/plants13223248)
Supplement: Supplementary file 1 [file plants-13-03248-s001.zip › TableS3.pdf]

Table S3. Morphological characteristics of *C. annuum* plants (CHMX_Ch1 and QO).

**Parameter**	**CHMX_Ch1**	**QO**
Locality	Chínipas, Chihuahua	El Patol, Querétaro
Coordinates	27°24'0" N, 108°32'0" W; 555 masl	20°44'8" N, 99°51'45" W; 2,258 masl
Stem diameter	3.707 ± 0.43	Data not available
Plant canopy	11.33 ± 3.06	Data not available
Plant height	42.00 ± 5.29	Data not available
Leaf shape	Deltoid (triangular)	Data not available
Leaf length	3.857 ± 0.82	Data not available
Leaf width	2.536 ± 0.52	Data not available
Pubescence density	High	Data not available
Seed texture	Rough surface	Data not available
Seed size	3.004 ± 0.36	Data not available
Seed width	0.766 ± 0.08	Data not available
Growth climate	Warm – semi-arid	Semi-arid
Annual precipitation (mm)	781.7	528
Annual average temperature (°C)	23.8	30.7

Morphological data for the QO genome are not available in the literature and are indicated as "Data not available" in the table.
